# Asymmetric Relationship between Ambient Air Temperature and Incidence of COVID-19 in the Human Population

**DOI:** 10.4269/ajtmh.21-0328

**Published:** 2022-01-28

**Authors:** Moiz Usmani, Yusuf Jamal, Mayank Gangwar, Bailey Magers, Juan Chaves-Gonzalez, Chang-Yu Wu, Rita Colwell, Antarpreet Jutla

**Affiliations:** ^1^ GeoHealth and Hydrology Lab (GeoHLab), Department of Environmental Engineering Sciences, University of Florida, Gainesville, Florida;; ^2^ United Nations Office for the Coordination of Humanitarian Affairs, New York, New York;; ^3^ Department of Environmental Engineering Sciences, University of Florida, Gainesville, Florida;; ^4^ University of Maryland Institute for Advanced Computer Studies, University of Maryland, College Park, Maryland

## Abstract

The complexity of transmission of COVID-19 in the human population cannot be overstated. Although major transmission routes of COVID-19 remain as human-to-human interactions, understanding the possible role of climatic and weather processes in accelerating such interactions is still a challenge. The majority of studies on the transmission of this disease have suggested a positive association between a decrease in ambient air temperature and an increase in human cases. Using data from 19 early epicenters, we show that the relationship between the incidence of COVID-19 and temperature is a complex function of prevailing climatic conditions influencing human behavior that govern virus transmission dynamics. We note that under a dry (low-moisture) environment, notably at dew point temperatures below 0°C, the incidence of the disease was highest. Prevalence of the virus in the human population, when ambient air temperatures were higher than 24°C or lower than 17°C, was hypothesized to be a function of the interaction between humans and the built or ambient environment. An ambient air temperature range of 17 to 24°C was identified, within which virus transmission appears to decrease, leading to a reduction in COVID-19 human cases.

## INTRODUCTION

On December 27, 2019, a local hospital in Wuhan, Hubei province, China, notified the Chinese Center for Disease Control and relevant health commissions of a mysterious pneumonic disease cluster. Thus, began the global COVID-19 pandemic caused by severe acute respiratory syndrome coronavirus-2 (SARS-CoV-2), as declared by the WHO on March 11, 2020. As of September 2021, COVID-19 infection was reported in more than 200 countries and territories, with an average of 4.88 individuals infected each second and 4.7 deaths every minute. Although the etiological pathways of COVID-19 are still emerging, previous studies of coronavirus (notably SARS-CoV-1) human cases in various geographic locations from 2002 to 2004 reported that coronavirus viability and survival on surfaces depended on a combination of temperature and relative humidity.^1^^,^^2^ Recent publications^3^^–^^5^ suggest a possible route of transmission of the virus occurring via aerosols. Wang and Du^5^ reported individuals becoming infected without contact with known confirmed human cases. Previous laboratory studies have determined that dry air conditions during low ambient air temperatures (with low dew point temperatures) were directly proportional to survival of aerosolized virus.^6^^–^^11^ An interesting finding, shown in Supplemental Table 1, is that almost all the studies had suggested a positive association between cold ambient temperature and incidence of COVID-19 in the human population. Yet, observations based on actual COVID-19 occurring in humans at different locations (e.g., within the United States, India, China) indicate that warm temperature may have a similar impact on prevalence of the disease, thus creating a paradox. It raises a critical question concerning the role and relationship of ambient air and dew point temperatures and incidence of COVID-19, which comprise the motivation for this study. Although rapid transmission of SARS-CoV-2 can be attributed to human-to-human interactions,^12^^,^^13^ the role of the ambient and built environment, as well as climate and weather processes, in enabling transmission conditions of the virus is not fully understood. Therefore, the objective of this study was to provide a data-driven adaptive assessment on: 1) association of ambient air and dew point temperature with respect to the incidence of COVID-19 in the human population and 2) development of a generalizable heuristic (across geographic boundaries) hypothesis using the information derived and validated from early epicenters of this disease. Resolution of these issues will be helpful in developing an experimental climate-informed hypothesis translatable to a predictive model for estimating the risk of infection in humans.

## METHODS

We examined the association of ambient air and dew point temperatures with the incidence of COVID-19 in the human population by employing time series analysis. The literature suggests symptoms for COVID-19 begin to appear in humans within a median range of 4.1 days of exposure,^14^ and therefore, for this study, we used a 4-day running average for air and dew point temperatures. Parametric (Pearson) and nonparametric (Kendall-Tau) cross-correlations were used to determine the associational linkages between ambient air and dew point temperature with reported COVID-19 cases. We validated statistically the association of temperature with COVID-19 cases using relative risk ratio (RR), the ratio of the probability of presence and probability of absence in a specific scenario. Finally, k-means cluster analysis was used to quantify the effect of nonlinearity between ambient air temperature and reported COVID-19 cases.

Data on ambient air temperature and dew point temperature were obtained from the NASA’s Modern-Era Retrospective Analysis for Research and Applications Version 2 (MERRA-2) at a resolution of 0.5° × 0.625°. Parameter elevation Regression on Independent Slopes Model (PRISM Climate Group, Oregon State University, http://prism.oregonstate.edu) data, at 4-km by 4-km resolution, obtained from Oregon State University. Data on daily COVID-19 cases in U.S. counties were downloaded from an open source upstream repository (https://github.com/datasets/covid-19) maintained by Johns Hopkins University Center for Systems Science and Engineering (CSSE). Dew point temperature was defined as the temperature at which water vapor condenses to form liquid droplets and the droplets are large enough to settle quickly on the surface of the earth due to gravity. The dew point temperature value cannot exceed ambient air temperature, and the dew point must be interpreted within the context of ambient air temperature. The difference between ambient air temperature and dew point temperature is an indicator of moisture availability in the atmosphere.^7^^,^^8^ For example, a difference of 20°C at lower and higher air temperatures will have a different significance in terms of moisture content and vapor saturation in the air. Lower dew point temperature implies less moisture in the air, that is, the lower the dew point temperature, the more likely a virus particle will remain afloat in ambient air because of absence of water droplets.^6^^–^^11^ During summer months, dew point temperatures are higher than in winter months, indicating more air moisture in summer season. Thus, in winter months, a difference of 20°C compared with summer months suggests less moisture (in terms of weight and volume) in the air, contributing to viruses remaining suspended in the air.^6^^–^^11^

## RESULTS

Four epicenters of COVID-19 (Daegu, South Korea; Munich, Germany; Tehran, Iran; and Wuhan, China) were selected to determine the ambient and dew point temperature relationship with temperature COVID-19 cases. On the basis of evidence from the literature, symptoms for COVID-19 begin to appear in humans within a median range of 4.1 days of exposure.^14^ Therefore, the analysis used an average of 4 days before known index cases being reported in a region to determine the association of air and dew point temperatures. The goal was to establish if it was possible to identify climatic and weather patterns occurring before time of the report, based on best available information, of human index case(s).

**Figure 1.**
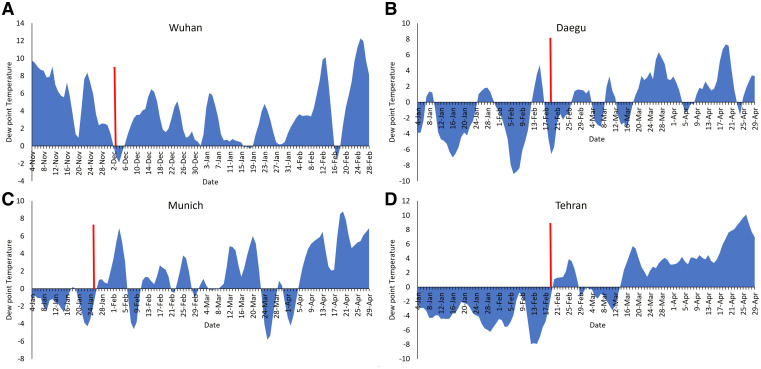
Four-day average dew point temperatures in (**A**) Wuhan, (**B**) Daegu, (**C**) Munich, and (**D**) Tehran. Red (vertical) line indicates the best information for initiation of an outbreak or first reported case. This figure appears in color at www.ajtmh.org.

**Figure 2.**
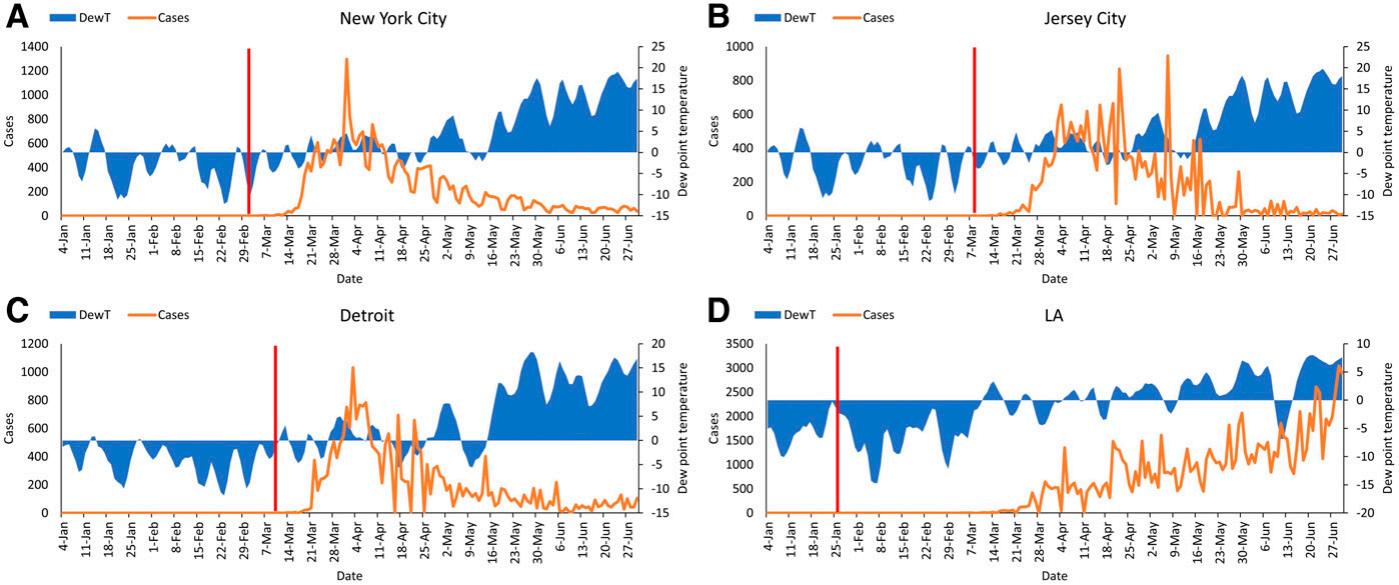
Four-day average dew point temperatures for (**A**) New York City, (**B**) Jersey City, (**C**) Detroit, and (**D**) Los Angles. Red (vertical) line, as shown, indicates the best information for beginning of the outbreak or first reported case. This figure appears in color at www.ajtmh.org.

In December 2019, the first case of COVID-19 was reported (approximately December 1, 2019) in Wuhan, China, and an exponential rise in the number of human cases quickly followed.^15^ In late November and early December 2019, Wuhan experienced a negative 4-day average value in dew point temperature (Figure 1A), and cases began to increase thereafter (a reliable time series of human cases is not available; hence, the heuristic argument is presented here). Daegu is a city in North Gyeongsang province of South Korea and was the epicenter of COVID-19 in that country. The city faced an exponential increase in number of reported cases in February 2020 and experienced a 4-day average subzero dew point temperature (Figure 1B). The other two outbreak epicenters included in this study (Tehran, Iran, and Munich, Germany) revealed consistent climatic conditions (Figure 1)—namely, negative dew point temperature prior to the COVID-19 outbreak (Figure 1). Because COVID-19 prevalence time series were available in the United States, four major outbreak centers (New York City, NY; Jersey City, NJ; Detroit, MI; and Los Angeles, CA) were selected for analysis. Beginning January 17, 2020, New York City did not record a positive dewpoint temperature for several consecutive days (Figure 2A), instead it had negative dew point temperatures until the end of May 2020. For the first 3 months of 2020, these four U.S. cities experienced below 0°C dewpoint temperatures (Figure 2). Figure 2 shows an approximate best available date (indicated by the red line) when the first index cases were reported for these four cities, which was during the time period when dew point temperatures were below 0°C.

Major outbreaks before May 2020 were concentrated in regions where air and dew point temperatures were less than 0°C. However, a rapid increase in geographic reach of this virus became evident when by October 2020, almost all countries (≈180) reported outbreak of COVID-19 in human population. This nullified, to some degree, that the virus is prevalent only in low ambient air and dew point temperature regions, as had been reported in previous studies.^5^^,^^16^^,^^17^ To determine the impact of ambient air temperature, it is necessary to distinguish between cold and warm regions. Cold regions, for the purpose of this study, were defined as those where the daily average ambient air temperature was below 0°C for ≥ 3 consecutive months in a year, irrespective of number of days each month. All others were classified as warm regions. For this analysis, 15 additional major epicenters in the United States were selected based on the availability of fine spatial scale climatic and disease datasets.

Cold regions (Illinois, Massachusetts, New Jersey, New York, and Pennsylvania) reporting COVID-19 cases had a peak in the number of reported cases before the end of May 2020 (Supplemental Figure 1). After a stay-at-home or “lockdown” directive (Supplemental Figure 1), U.S. counties and cities had begun to open for business around May 2020.^18^ By September 2020, highly populated counties in warm regions (Arizona, California, Florida, Georgia, and Texas) had reported higher numbers of new cases than cold regions (Supplemental Figure 2). Demarcation of the impact of cold and hot regions is evident from the time series of COVID-19 cases (Supplemental Figure 3). Positive case rates were higher during colder than warmer months. An observation to note from Supplemental Figures 1 and 2 is that, despite lockdown, cold regions experienced more cases during colder months than warm regions. Overall, the rate of increase in number of cases during winter months was greater for cold region counties (0.3 relative cases per day between March 19, 2020 to April 8, 2020) than summer months in warm regions (0.15 relative cases per day between June 18, 2020 to July 9, 2020) (Supplemental Figure S4).

Whereas there appears to be a seasonal basis for separation of boundaries between cold and warm regions with respect to the occurrence of cases in the human population, it remains to be determined whether there is a temperature range within which the number of COVID-19 cases decreases. The human ergonomics literature suggests the tolerable ambient air temperature for human wellness is 17–24°C.^19^^–^^22^ This temperature range encourages outdoor activities and less reliance on mechanical air conditioning, with positive or negative deviation being associated with increase in indoor activity. Cross-correlation analysis between maximum air temperature and number of COVID-19 cases for major epicenter cities in the United States was conducted (Table 1). The result revealed that warm regions where ambient air temperatures are > 24°C show a statistically significant correlation with COVID-19 cases, similarly for cold regions (Table 1).

**Table 1:** Correlation between reported cases and maximum daily temperature in warm and cold regions: *n* = number of days for each location (for some locations the number of cases was more than 5,000 per day)

Cities	Pearson (*N* = 219)	Kendall tau (*N* = 219)
Warm		
Atlanta	0.63*	0.58*
Dallas	0.62*	0.65*
El Paso	0.66*	0.67*
Houston	0.60*	0.64*
Jacksonville	0.51*	0.57*
Los Angles	0.68*	0.51*
Miami	0.46*	0.56*
New Orleans	0.20*	0.40*
Phoenix	0.68*	0.70*
Cold		
Chicago	−0.67*	−0.43*
Detroit	−0.53*	−0.39*
Jersey City	−0.61*	−0.43*
New York City	−0.70*	−0.65*

* Statistically significant correlation at 95% confidence interval.

**Figure 3.**
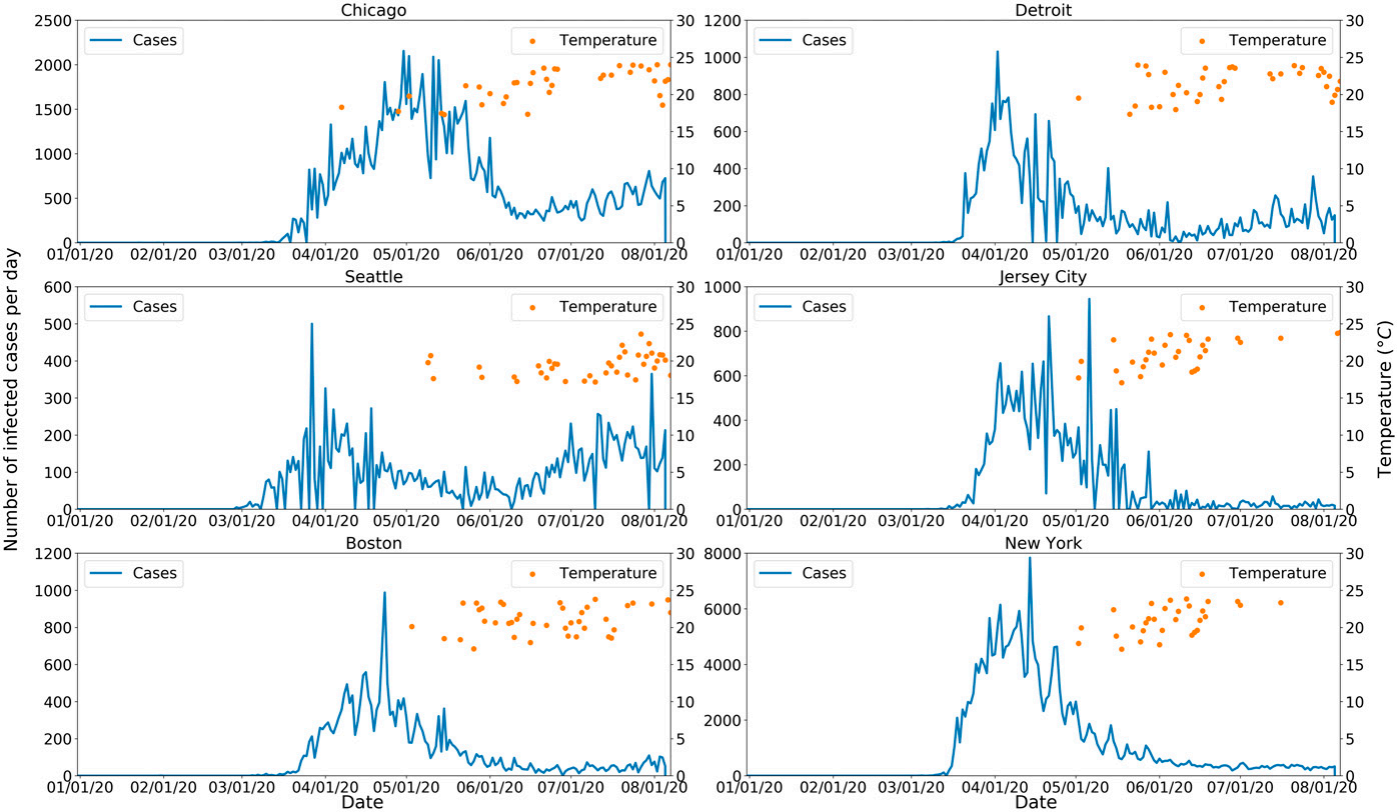
COVID-19 cases (line) and tolerable temperature (dots) in six major cold region epicenters in the United States. Only those dates when the temperature was within the tolerable range are provided for purposes of clarity. This figure appears in color at www.ajtmh.org.

**Figure 4.**
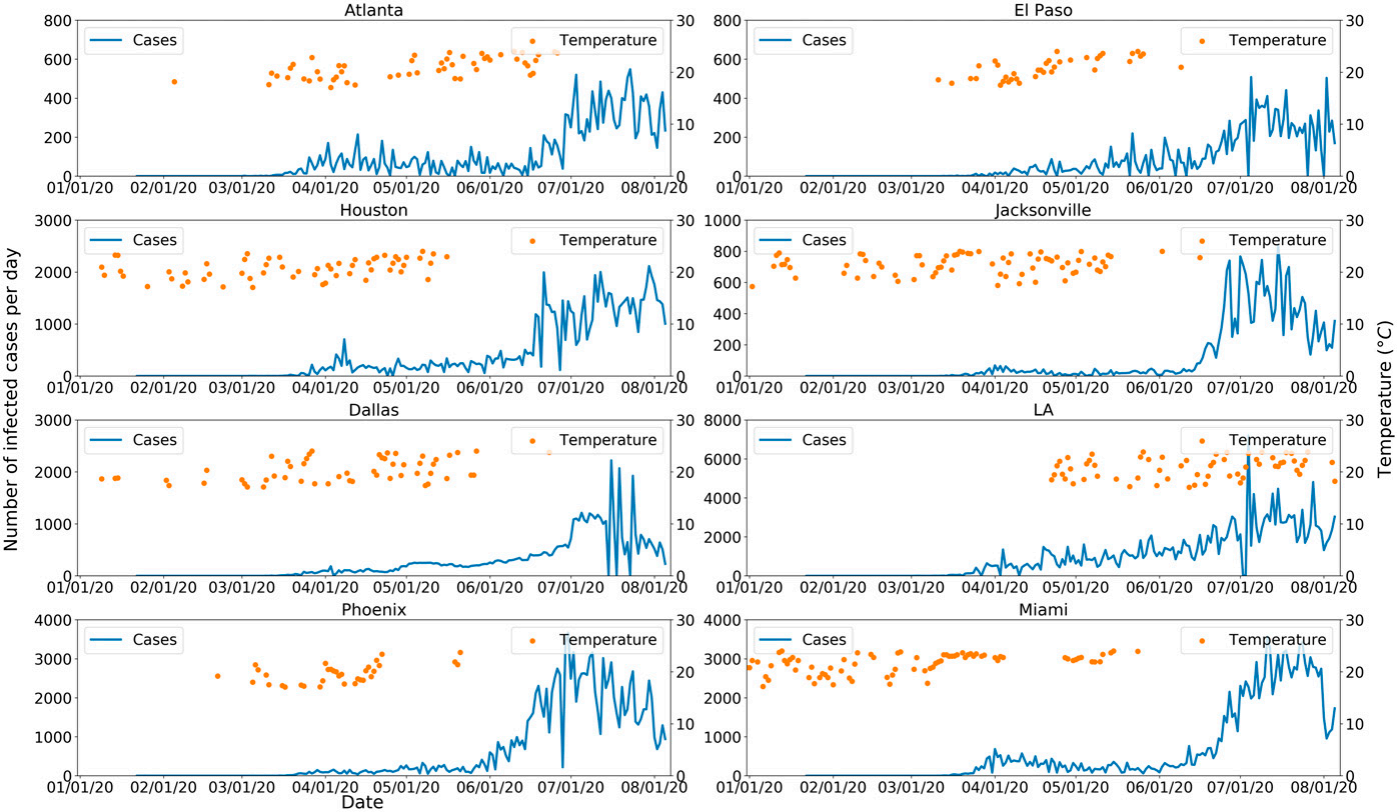
COVID-19 cases (line) and tolerable temperature (dots) for eight warm region epicenters in the United States. Only those dates when the temperature was within the tolerable range are shown for purposes of clarity. This figure appears in color at www.ajtmh.org.

**Figure 5.**
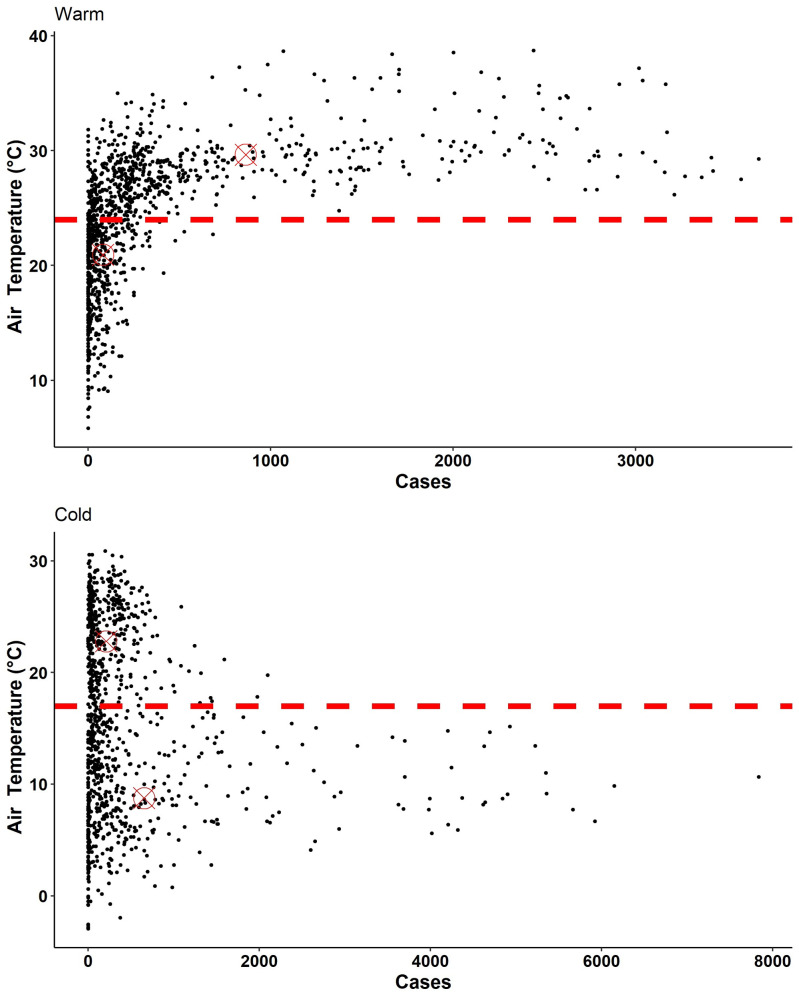
Clustering of disease prevalence vs ambient air temperature. (**A**) Clustering for warm regions. (**B**) Clustering for cold regions. Red (horizontal) line in the warm region represents 24°C ambient air temperature and in cold region 17°C. This figure appears in color at www.ajtmh.org.

Temperatures of 17–24°C and the number of COVID-19 cases for six major cold region epicenters of outbreaks are shown in Figure 3 for March and April 2020. The results show temperatures within this range are associated with a decrease in number of COVID-19 cases, except for Seattle, WA, where the overall number of human cases remained relatively low, when compared with other locations of the analysis. Figure 4 shows tolerable temperature ranges for warm region epicenters, suggesting an increase in cases is observed when ambient air temperatures are outside the 17–24°C temperature range. A nonlinear relationship between temperature and COVID-19 cases having been observed, RR analysis was conducted to quantify the association of temperature and COVID-19 human cases. Table 2 shows RRs for average number of cases at temperatures > 24°C (warm regions) or < 17°C (cold regions). Here the risk ratio is the ratio between the probability of higher than the average number of COVID-19 cases during temperatures outside the 17–24°C range to the probability of higher than the average number of cases when temperatures are between 17 and 24°C. For a warm region (e.g., Atlanta, GA), the RR for average cases is 4.03, implying the odds of occurrence of an average number of cases (125) when the ambient air temperature is warmer than 24°C is 4.03 times higher than when the temperature is lower than 24°C. For Jacksonville, MS; Dallas, TX, Miami, FL; and Phoenix, AZ, where the probability of absence was zero, it was concluded that viable RRs cannot be computed and indicated COVID-19 cases in these locations would always exceed the average number of observed cases (zero odds of absence). A similar observation was noted for cold regions (Chicago, IL; Detroit, MI; Jersey City, NJ; Boston, MA; New York, NY), except Seattle, when temperatures were less than 17°C. Confirmation was obtained using k-means cluster analysis,^23^ the basic premise of which is to detect discernable clusters (hence boundary of separation) detectable between cold and warm temperatures for both regions. In warm regions (Figure 5A), a large number of cases were clustered at high temperatures than otherwise, with the opposite for cold regions (Figure 5B).

**Table 2:** Relative risk ratio of COVID-19 cases in the temperature range > 24° or < 17°C; i.e., outside the tolerable range with respect to COVID-19 cases occurring within the tolerable temperature range (> 17° and < 24°C)

	Average cases	Probability in favor	Probability in absence	RRs
Warm region				
Atlanta	125	0.44	0.11	4.03
Jacksonville	147	0.39	0.00	–
New Orleans	69	0.24	0.20	1.19
El Paso	101	0.35	0.03	10.19
Dallas	345	0.53	0.00	–
Miami	834	0.43	0.00	–
Phoenix	810	0.57	0.00	–
Los Angeles	1,314	1.00	0.92	1.09
Houston	535	0.53	0.02	33.80
Cold region				
Chicago	713	0.44	0.17	2.67
Detroit	181	0.33	0.04	7.44
Seattle	104	0.24	0.45	0.53
Jersey	127	0.37	0.04	10.34
Boston	141	0.38	0.00	–
New York	1,483	0.60	0.00	–

RR = risk ratio.

## DISCUSSION AND CONCLUSION

**Figure 6.**
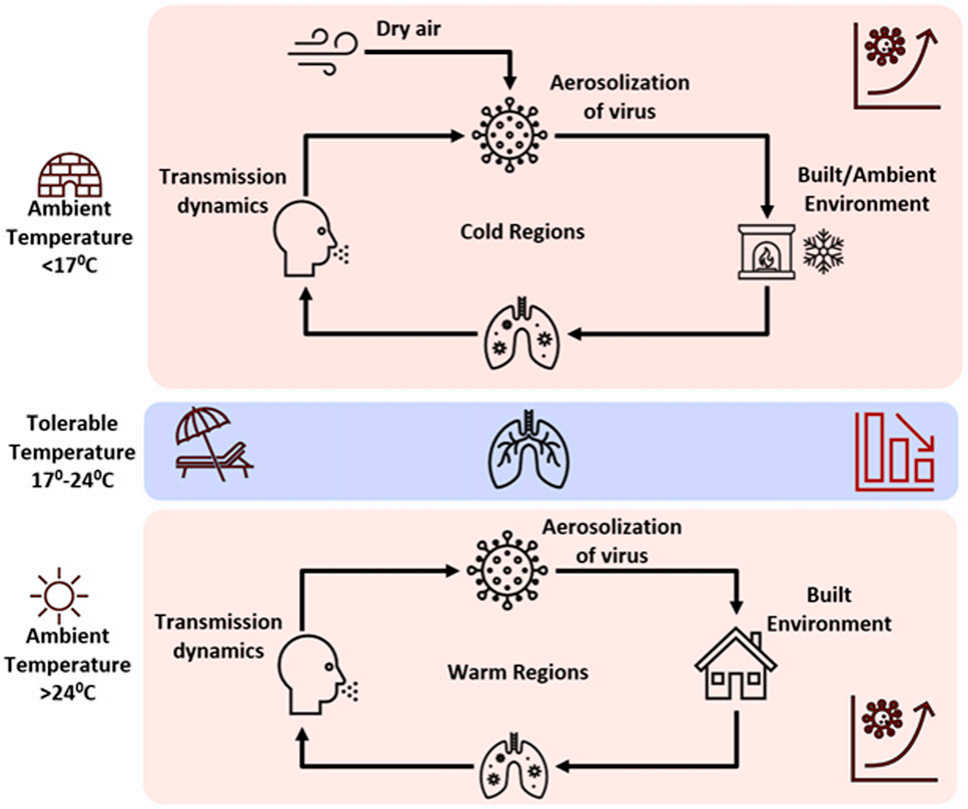
Hypothesis for COVID-19 infection. This figure appears in color at www.ajtmh.org.

The objective of this study was to determine the association of ambient and dew point temperature with incidence of COVID-19. On the basis of heuristic data analysis, a tolerable temperature range of 17–24°C was determined that corresponded to decrease in number of COVID-19 cases in the human population. However, a correlative analysis may not be concluded as causative. Therefore, a generalizable hypothesis governing the dynamics of COVID-19 disease in a human population is proposed (Figure 6). It provides a mechanistic interpretation of the role temperature (both ambient air and dew point) plays and relies on potential aerosolization of the virus via particulates in the ambient and built (indoor) environment. Lack of moisture in ambient air promotes aerosolization of the virus and suspension of particles in air over a longer time periods.^24^^,^^25^ Aerosolization of the virus in the built environment has been reported,^4^^,^^24^^–^^26^ and its presentation in ambient air is currently under study. Ambient air with a very low dew point temperature is used to support aerosolization^6^^–^^11^ of the virus, and once aerosolized, SARS-CoV-2 can be transmitted in both in built and ambient environments in cold regions. Dew point temperature is proportional to moisture in the air; the lower the dew point temperature, the lower the moisture content of the air. Dew point temperature in cold regions from March 11, 2020, to the end of June 2020 showed that case prevalence had a significant (*P* < 0.001) negative correlation with dew point temperature, with a lag of 2 to 3 weeks (Table 3). An ambient temperature range of 17–24°C is that within which the number of COVID-19 cases decreases in cold (Figure 3) and warm (Figure 4) regions. However, an extremely low or high air temperature had a similar effect on the number of COVID-19 cases in the built environment. Both extremes of ambient temperatures are associated with human activity shifting indoors, promoting exposure to recirculated air. In contrast, cold temperature contributes to ambient air aerosolization of the virus, resulting in more human COVID-19 cases. It can be argued that, in warm regions, there will be a decrease in aerosolization of virus in the ambient environment, although research is needed to confirm this supposition. Nevertheless, the literature on human ergonomics suggests that as the temperature increases above a specific threshold (generally 24°C), humans tend to spend time indoors and in a mechanically controlled environment.^27^ The latter will result in 1) drying of the indoor air, making the indoor environment suitable for aerosolization, or 2) enhancing close interaction with infected (perhaps asymptomatic) individuals, leading to COVID-19 transmission. There is strong evidence that community transmission of coronavirus increases in closed environments^4^^,^^28^^–^^30^ and, in several instances, a few infected humans caused significant outbreaks.^31^^–^^33^ Recent examples of indoor congregating in warm regions resulted in COVID-19 outbreaks, and these included religious gatherings in India, Pakistan, Malaysia, and South Korea.^34^^,^^35^

**Table 3:** Correlation between reported cases and dew point temperature in cold region epicenters

Cities	Pearson (lag) (*N* = 112)	Kendall tau (lag) (*N* = 112)
Chicago	−0.56 (17)	−0.41 (17)
Detroit	−0.44 (12)	−0.35 (12)
Jersey City	−0.44 (9)	−0.33 (9)
New York City	−0.52 (9)	−0.45 (9)
Seattle	−0.44 (20)	−0.29 (20)

A complementary analysis to validate the hypothesis included four epicenter cities—Bengaluru, New Delhi, Pune, and Ludhiana of the 2021 COVID-19 India. These four cities closely resemble warm regions in the United States. The hypothesis is that similar climatic patterns should prevail before an increase in COVID-19 cases in these cities. Figure 7 shows that all began experiencing ambient air temperatures > 24°C during the first week of March 2021, and this increase was immediately followed by a surge in number of COVID-19 cases during the second and third weeks of March. Figure 7 shows that these locations exhibited a similar pattern of increase in the number of cases with an ambient air temperature outside the 17–24°C range.

**Figure:**
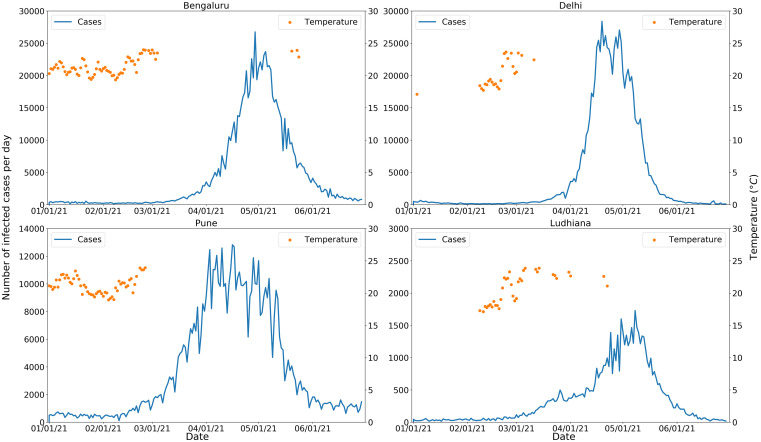
Figure 7. Cases of COVID-19 and ambient air temperature for four epicenters in India. This figure appears in color at www.ajtmh.org.

On the basis of data to August 2020 and the hypothesis, as shown in Figure 6, results should be independent of the time horizon; hence, further complementary analysis included March 8, 2020, to August 8, 2021. RR analysis as can be seen in Table 4 shows all epicenters, except for Miami, with risk ratios greater than 1. That is, if the temperature range is either less than 17°C or greater than 24°C, the region will have a higher probability of occurrence of COVID-19 than average cases reported in the region. A pattern of decrease in number of COVID-19 cases when the ambient temperature is within the range of 17–24°C and increase when the temperature is outside this range, strongly suggesting seasonality in COVID-19 in both warm and cold regions. For example, during winter, if a region experiences ambient air temperatures below 17°C, an increase in COVID-19 cases would be expected. Similarly, during summer months, if the ambient air temperature is higher than 24°C, an increase in COVID-19 cases would be expected. If cold regions do not experience temperatures > 24°C, they should expect high numbers of cases in the winter. On the other hand, if a warm region does not experience temperatures < 17°C during the winter months, a peak in cases would occur only during summer. Regions that experience both, temperature extremes (i.e., > 24°C and < 17°C) will likely have two seasonal peaks of COVID-19 cases.

**Table 4:** Relative risk ratio of COVID-19 cases in the temperature range > 24° or < 17°C*

	Average cases	Probability in favor	Probability in absence	RRs
Warm region				
Atlanta	206	0.33	0.06	6.06
Jacksonville	268	0.30	0.13	2.20
New Orleans	75	0.24	0.19	1.29
El Paso	267	0.24	0.23	1.02
Dallas	626	0.37	0.15	2.48
Miami	1,117	0.35	0.63	0.56
Phoenix	1,150	0.32	0.22	1.49
LA	2558	0.45	0.34	1.32
Houston	854	0.38	0.27	1.40
Cold region				
Chicago	1,097	0.52	0.17	3.11
Detroit	327	0.33	0.08	4.34
Seattle	235	0.30	0.11	2.58
Jersey	173	0.35	0.09	3.77
Boston	187	0.34	0.05	7.19
New York	656	0.28	0.08	3.27

RR = risk ratio.

* Outside the tolerable range with respect to COVID-19 cases occurring within the temperature range > 17° and < 24°C, within the tolerable range from March 8, 2020 to August 11, 2021.

An exception was noted for Miami, where an increase in number of cases between October 2020 and February 2021 was observed, despite the region having comfortable temperatures (> 17°C and < 24°C). However, only Miami showed a positive trend—namely, an increase over time in the three modes of mobility (driving, transit, and walking), as shown in Supplemental Figure 5. During the COVID-19 outbreak, anomalous resettlement of population from northern (cold) regions was reported in Miami (https://www.miamiherald.com/news/business/article246712596.html), which corroborates with the increased mobility patterns of Supplemental Figure 5. With rapid movement, there is an inherent risk of virus transmission, accelerating human-to-human interactions in the population. In the absence of changes in mobility pattern (Supplemental Figure 5), the modalities of built and ambient air and dew point temperature are likely to contribute to the transmission of COVID-19. It is concluded from the results that aerosolized viruses may be correlated with the climate modalities of dewpoint and ambient temperature, taking into account that aerosolized viruses behave differently indoors and outdoors.^36^ Various models have been developed to simulate air filtration systems, circulation, social distancing, and use of face masks, all of which can affect the length of time aerosols remain suspended and the distance traveled.^37^^–^^39^ Human-to-human transmission remains a critical and dominant pathway for spread of the virus (Figure 6). The results of this study strengthen the argument that once the virus is introduced, climatic conditions—namely, temperature—play an important role in accelerating the spread of the virus. Although the infection dose of aerosolized virus remains to be precisely determined, the aerosol transmission of COVID-19 is an important mode of spread in both warm and cold regions.

## Supplemental Material


Supplemental materials
