# Supplementary material for: Asymmetric Relationship between Ambient Air Temperature and Incidence of COVID-19 in the Human Population
Source: Am J Trop Med Hyg. 2022 Jan 28;106(3):877–85. doi: 10.4269/ajtmh.21-0328 (PMC8922505; doi:10.4269/ajtmh.21-0328)
Supplement: Supplementary file 1 [file tpmd210328.SD1.pdf]

Supplementary Figures

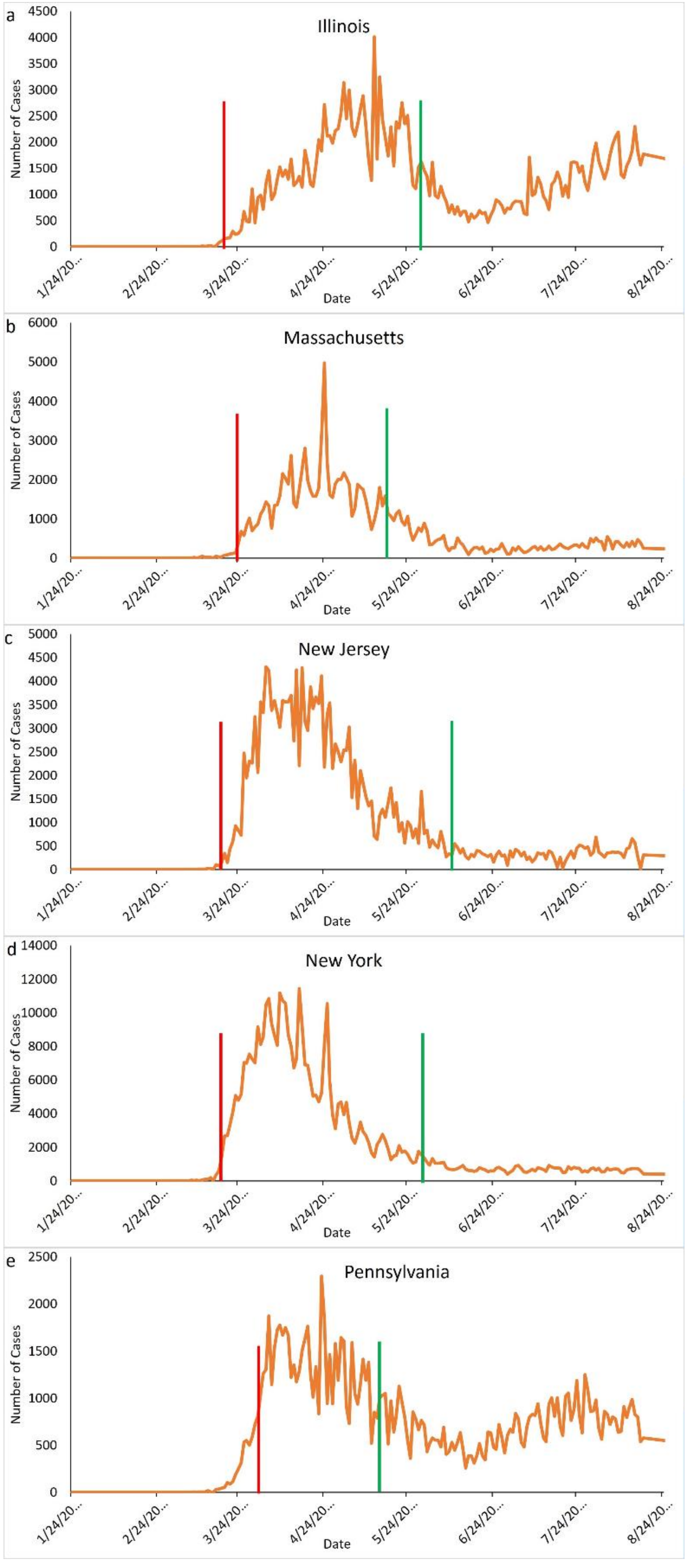

Figure S1: Duration of lockdowns in colder states. Red (first vertical) line marks beginning of lockdown in the State and green (second vertical) line marks end of lockdown.

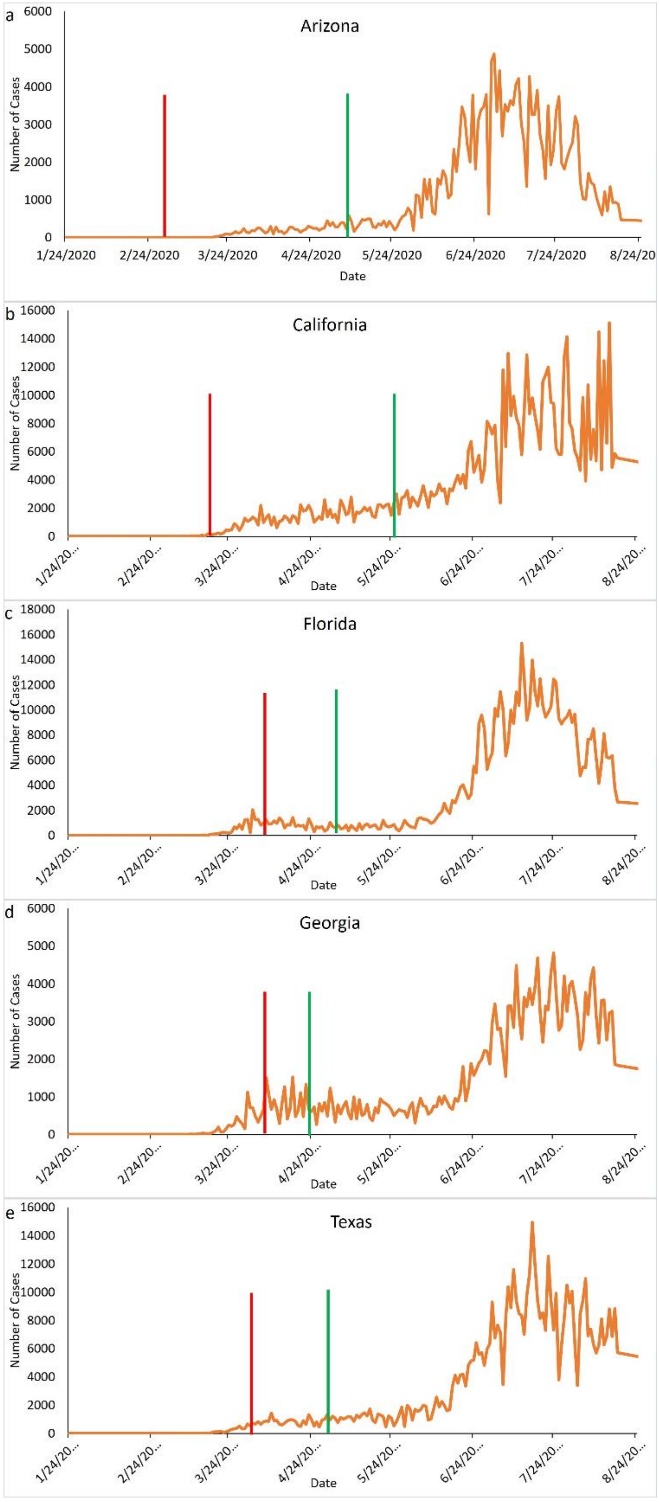

Figure S2: Duration of lockdowns in warmer states. Red (first vertical) line marks beginning of lockdown in the State and green (second vertical) line marks end of lockdown.

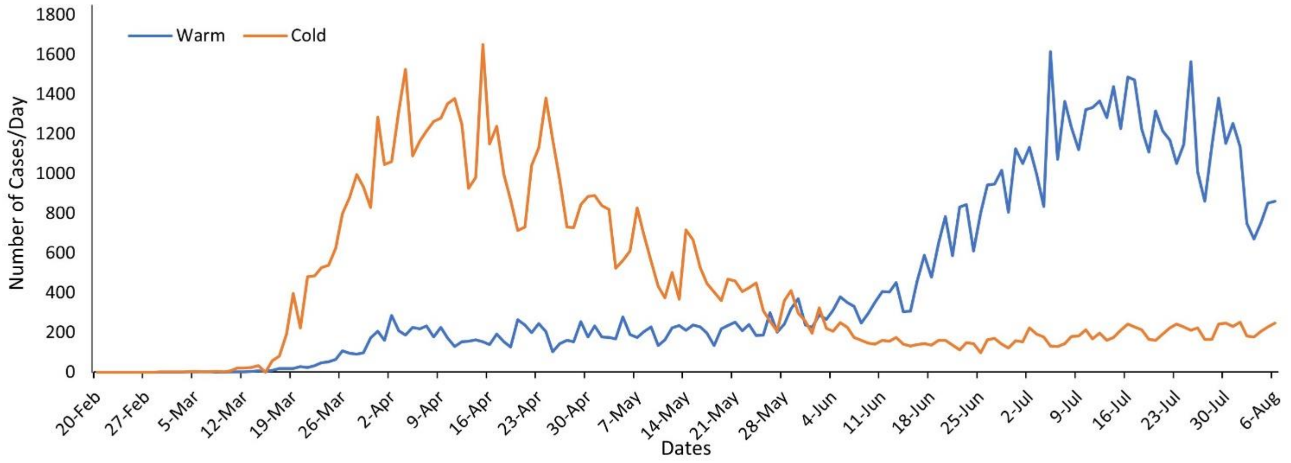

Figure S3: Time series of total infected cases per day in cold and warm regions of the United States of America (Year 2020 only).

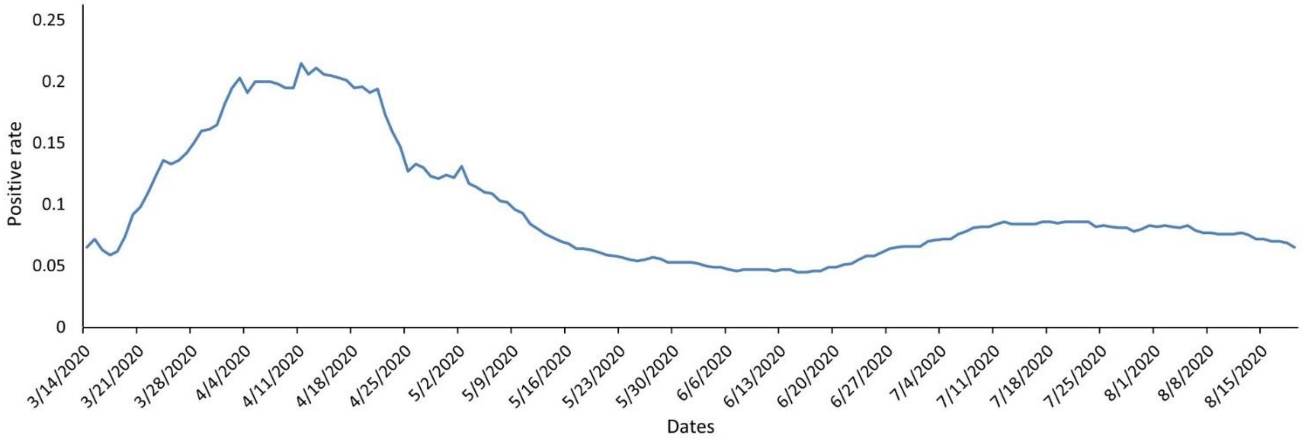

Figure S4: Time series for positivity rate of COVID-19 tests in the United States (number of positive cases reported divided by total number of tests conducted).

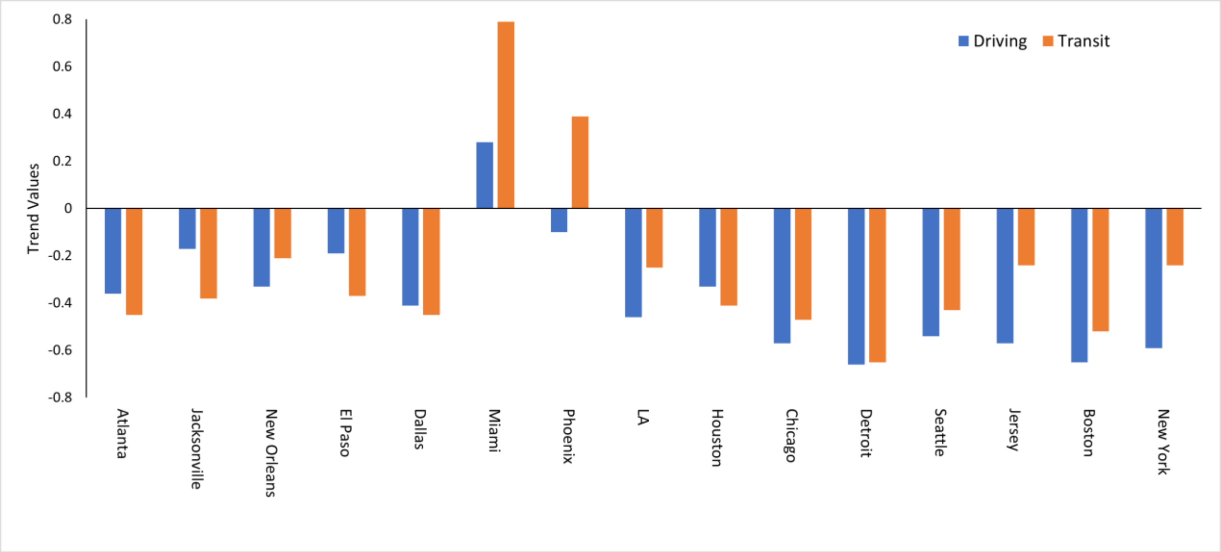

Figure S5: Trend of two modes of mobility between September 2020 and February 2021. Trend is defined as the prevailing tendency of a variables with respect to time, which is calculated through the correlation between the variable and the time period.

Supplementary Table

Table S1: Environmental association of COVID-19 and influenza

#	Paper	Authors	Citation	Summary	Location
**COVID-19**					
1	Association between ambient temperature and COVID-19 infection in 122 cities from China	Xie, J. and Zhu, Y.	239	Association with low temperature and no evidence for warm temperature	122 cities in China
2	A spatio-temporal analysis for exploring the effect of temperature on COVID-19 early evolution in Spain	Briz-Redon, A. and Serrano-Aroca, A.	78	No evidence of relationship	Spain (Iberian Peninsula)
3	Asymmetric nexus between temperature and COVID-19 in the top ten affected provinces of China: A current application of quantile-on-quantile approach	Shahzad et al	30	No conclusive result	China (10 Provinces)
4	Temperature significantly changes COVID-19 transmission in (sub)tropical cities of Brazil	Prata et al	133	Negative association at temperature between 16.8C to 25.8C.	Brazil (27 cities)
5	Impact of temperature on the dynamics of the COVID-19 outbreak in China	Shi et al	86	Negative association and peaks at 10C	China
6	High Temperature and High Humidity Reduce the Transmission of COVID-19	Wang et al	309	Negative association with reproductive number	China and USA
7	Effects of temperature variation and humidity on the death of COVID-19 in Wuhan, China	Ma et al	301	Positive association with death in Wuhan.	Wuhan, China
8	Temperature, Humidity and Latitude Analysis to Predict Potential Spread and Seasonality for COVID-19	Sajadi et al	264	5-11 C temperature range region are at high risk.	8 cities (Wuhan, Tokyo, Daegu, Qom, Mila, Paris, Seattle, and Madrid)
9	Effects of temperature and humidity on the daily new cases and new deaths of COVID-19 in	Wu et al	141	Negative association	166 countries (excluding China)

	166 countries				
10	COVID-19 transmission in Mainland China is associated with temperature and humidity: A time-series analysis	Qi et al	144	Negative association	China
11	The sensitivity and specificity analyses of ambient temperature and population size on the transmission rate of the novel coronavirus (COVID-19) in different provinces of Iran	Jahangiri et al	46	High sensitivity to air temperature	Iran
**Influenza**					
1	Influenza Virus Transmission Is Dependent on Relative Humidity and Temperature	Lowen et al	1393	Cold temperature favors transmission	
2	Absolute Humidity, Temperature, and Influenza Mortality: 30 Years of County-Level Evidence from the United States	Barreca	163	Cold temperature favors transmission	United States
3	Effects of temperature, humidity, and diurnal temperature range on influenza incidence in a temperate region	Park et al	51	Significant increase at low temperature (0-5 C)	Seoul, Republic of Korea
4	Avian influenza virus in water: Infectivity is dependent on pH, salinity and temperature	Brown et al	200	Cold temperature favors transmission	
5	Decline in temperature and humidity increases the occurrence of influenza in cold climate	Jaakkola et al	94	Negative association at cold temperature	Finland
